# High-concentration continuous local antibacterial perfusion therapy: safety and potential efficacy for acute and chronic periprosthetic knee joint infection

**DOI:** 10.1051/sicotj/2024048

**Published:** 2024-11-26

**Authors:** Yuki Suzuki, Koji Iwasaki, Zenta Joutoku, Tomohiro Onodera, Masatake Matsuoka, Ryosuke Hishimura, Masanari Hamasaki, Eiji Kondo, Norimasa Iwasaki

**Affiliations:** 1 Department of Orthopaedic Surgery, Faculty of Medicine and Graduate School of Medicine, Hokkaido University Kita 15 jo, Nishi 7 chome, Kita-ku Sapporo Hokkaido 060-8638 Japan; 2 Department of Functional Reconstruction for the Knee Joint, Graduate School of Medicine, Hokkaido University Kita 15 jo, Nishi 7 chome, Kita-ku Sapporo Hokkaido 060-8638 Japan; 3 Department of Orthopaedic Surgery, Obihiro Kosei Hospital Nishi 14 jo, Minami 10 chome 1 Obihiro Hokkaido 080-0024 Japan; 4 Centre for Sports Medicine, Hokkaido University Hospital Kita 14 jo Nishi 5 chome, Kita-ku Sapporo Hokkaido 060-8648 Japan.

**Keywords:** Continuous local antibiotic perfusion, Total knee arthroplasty, Infection, Gentamicin, Periprosthetic joint infection

## Abstract

*Background*: Periprosthetic joint infections (PJIs) following total knee arthroplasty (TKA) are among the most challenging pathologies to manage. Recently, continuous local antibiotic perfusion (CLAP) therapy has been introduced for treating musculoskeletal infections in orthopedics. This study aimed to determine the outcomes and risks of CLAP therapy combined with conventional treatment for PJIs after TKA. *Methods*: We retrospectively evaluated 14 patients with PJIs. For acute PJIs, CLAP therapy was performed alongside debridement, intravenous antibiotics, and implant retention. For chronic PJIs, a two-stage revision with CLAP therapy and intravenous antibiotics was performed. Implants were replaced with a cement mold incorporating CLAP therapy, followed by revision surgery after 3 months. For all patients, 120 mg/day of gentamicin (GM) was locally administered into the knee joint for 2 weeks as part of CLAP therapy, in combination with perioperative intravenous antibiotics. *Results*: Five patients developed acute PJIs, and nine developed chronic PJIs after TKA. The mean follow-up period was 18.4 (15.2–21.1) months. All five patients with PJIs treated with one-stage surgery (debridement and insert exchange only) successfully preserved their implants. Among the nine patients with chronic PJIs, seven underwent CLAP therapy combined with two-stage revision surgery, resulting in successful treatment without relapse, whereas the remaining two patients were initially treated with one-stage surgery and CLAP therapy but failed to retain their implants, and subsequently required additional two-stage revision surgery, which ultimately succeeded. No adverse effects from GM were reported. *Conclusions*: Our results suggest that CLAP therapy is safe and may be effective for treating acute and most chronic PJIs after TKA.

## Introduction

Despite recent advancements in medicine, periprosthetic joint infections (PJIs) remain among the most challenging pathologies following arthroplasty. Estimates indicate that PJIs have a prevalence of 0.5%–1.9% after primary total knee arthroplasty (TKA) and 8%–10% after revision TKA [1–5]. PJIs after TKA lead to higher morbidity, longer hospital stays, and increased healthcare costs [6, 7]. Therefore, improved outcomes for PJIs after TKA are essential.

Managing PJIs requires complex treatment strategies, including multiple surgical interventions and long-term antimicrobial therapy. Surgical treatments have included debridement, intra venous antibiotics, and implant retention (DAIR), or the replacement of components through either a one- or two-stage procedures [8]. However, current findings indicate that up to 30% of patients develop recurrent infections [8]. Biofilm formation surrounding the implant [9, 10] and insufficient antibiotic concentrations at the infected site due to intravenous administration [11] have been identified as contributing factors to the low treatment success rate of PJIs.

Recently, a novel method of continuous local antibiotic perfusion (CLAP) has been developed for managing difficult infectious pathologies, such as open fractures and osteomyelitis [12, 13]. This treatment aims to maintain high local concentrations of antibacterial agents at the infection site. During CLAP therapy, high concentrations of antibiotics are continuously and directly injected into the local infected lesion, achieving sufficient localized antibacterial drug concentrations. Additionally, drainage can help reduce the risk of excessive increases in serum concentrations of antibiotics and minimize adverse effects. Although CLAP therapy is theoretically effective for PJIs after TKA, there are few reports regarding its outcomes in these cases. Therefore, this study aimed to clarify the short-term outcomes of CLAP therapy for acute and chronic PJIs after TKA.

## Materials and methods

### Patient recruitment

#### Ethics approval and consent to participate

This study was conducted in accordance with our institutional ethics policy. It received local institutional review board approval under protocol number 023-0033. Written informed consent was obtained from all patients for participation in this research, including the provision of patient information and any accompanying supplements for journal submission. Patients were provided a detailed explanation that this treatment involved off-label use of antibacterial drugs. The treatment was approved by the Certified Clinical Research Review Board of our hospital as an off-label use of aminoglycoside antibiotics. The procedures followed were in accordance with the ethical standards of the responsible committee on human experimentation (both institutional and national) and with the Helsinki Declaration of 1975, as revised in 2000.

#### Participants

Patients diagnosed with PJIs after TKA at our facility from May 2019 to June 2022 were retrospectively reviewed. The inclusion criterion was the ability to undergo 2 weeks of CLAP therapy. The exclusion criteria included a follow-up period of <6 months and patients with severe chronic kidney disease (CKD) [estimated glomerular filtration rate (eGFR) < 30 mL/min/1.73 m^2^] [14].

#### Diagnosis

PJIs were diagnosed according to the guidelines established by the American Academy of Orthopaedic Surgeons and the International Consensus Group on PJIs [15]. Patients who developed PJIs after TKA were categorized into two groups: (1) those with acute infection occurring within 1 month of the initial symptoms or diagnosis and (2) those with chronic infection occurring more than 1 month after the initial symptoms or diagnosis [8] regardless of the time since the previous surgery.

### Treatment protocol

#### Surgical treatment

Patients with acute PJIs underwent combination therapy that included DAIR, exchange of a polyethylene liner, and CLAP therapy (one-stage surgery). Meanwhile, patients with chronic infection typically underwent two-stage revision surgery with CLAP therapy and intravenous antibiotics, or one-stage surgery was performed based on the patient’s preference, provided there was no implant loosening. During the first stage of the two-stage surgery, we removed all materials and performed aggressive debridement, irrigation, and placement of antibiotic-loaded bone cement spacers followed by CLAP therapy and systemic antibiotic administration.

#### Antibiotic treatment

When PJIs were suspected, samples were collected prior to the administration of any antibiotics to maximize the likelihood of identifying the causative bacterial species. Based on our local prevalence of pathogens and their drug susceptibility patterns, we typically start with a first-generation cephalosporin (CEZ) targeting methicillin-sensitive *Staphylococcus aureus* (MSSA). However, depending on the results of rapid Gram staining, we may consider using sulbactam/ampicillin (SBT/ABPC) if *Bacillus* species are suspected. After evaluating bacterial identification and drug sensitivity from the collected samples, along with the patient’s systemic response to CEZ, we may switch to second-line antibiotics. Systemic antibiotics, that are sensitive to the detected bacteria were administered intravenously for 2 weeks, followed by at least 6 weeks of oral antibiotic treatment after surgery. Antibiotics were discontinued once clinical signs indicated no infection, such as fever, pain, redness, swelling, local heat, or elevation of any serum inflammation markers (white blood cell count, erythrocyte sedimentation rate, and C-reactive protein [CRP] level). For patients with chronic infection, the second-stage surgery (revision TKA) was performed after confirming no recurrence of infection following 4 weeks of antibiotic withdrawal and at least 12 weeks after the first-stage surgery.

#### CLAP therapy

**Figure 1:**
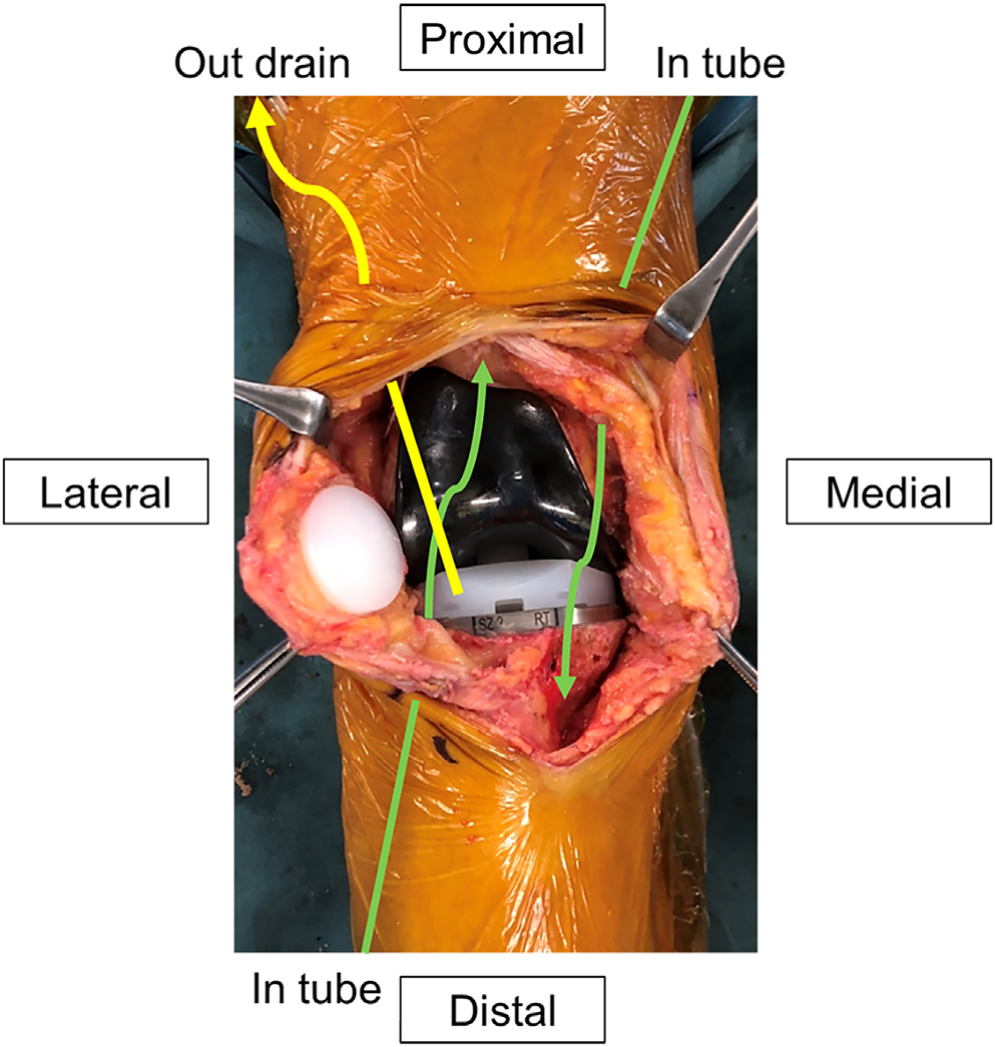
Scheme of the drain and tubes during continuous local antibiotic perfusion. The tips of the two drains were placed in the suprapatellar pouch and the posterior part of the tibiofemoral joint. The drainage tubes were positioned on the ventral side of the insert.

Equipment for CLAP therapy was introduced before wound closure during combined surgery. We have modified the previously reported CLAP method to make the treatment easier and more economical [12]. Two 5-Fr utility tubes (Atom Medical Corp, Tokyo, Japan) and a 15-Fr drain tube (Blake^®^ drains, Ethicon, Raritan, NJ) were inserted into the main wound (Figure 1). The two utility tubes were connected to a syringe pump, and a 15-Fr drainage tube was connected to the closed wound drainage system (J-VAC reservoirs^TM^, Ethicon). A high concentration of gentamicin (GM; 60 mg/saline 48 mL each) was continuously administered through the two inserted utility tubes at a rate of 2.5 mg/h (2 mL/h) using the syringe pump. The total amount of GM administered was 120 mg/day at the start of CLAP therapy. To ensure proper perfusion of the GM, the tips of the injection and drainage tubes were positioned far apart from each other. The tip of the utility tube was placed at the suspected main site of the infection, primarily in the suprapatellar pouch and the posterior part of the tibiofemoral (TF) joint. The 15-Fr drain was placed far from the utility tube, mainly on the ventral side of the TF joint space just posterior to the patella. The utility and drain tubes were secured to the skin: the former was fixed using two horizontal mattress sutures surrounding the tube, whereas the latter was secured by wrapping the ends of the simple drain hole suture around the tube and tightly tying it to prevent leakage.

CLAP therapy was performed for 2 weeks similar to the original method [12, 13]. Serum GM concentrations were monitored three times a week after surgery and were maintained below 2.0 μg/mL to prevent GM side effects. When GM concentrations exceeded 2.0 μg/mL, the amount of GM was reduced by 80 mg/day. If side effects of GM were observed, such as ototoxicity and renal dysfunction, CLAP therapy was discontinued immediately. Ototoxicity was assessed by an otolaryngologist before and immediately after CLAP therapy. During CLAP therapy, the knee was kept in extension and stabilized with a knee brace. Weight-bearing was prohibited for the affected extremity, and crutches or a wheelchair were used throughout the duration of CLAP therapy.

### Evaluation

#### Treatment and related outcomes

Follow-up details included clinical examinations, CRP measurements, and radiographic imaging every month for the first 3 months, followed by every 3 months thereafter. Successful treatment was defined as the absence of clinical symptoms, such as swelling, redness and local heat, along with negative CRP levels and no recurrence at the final follow-up.

#### CLAP therapy related outcomes

CLAP therapy-related outcomes, such as any drainage problems, serum and drain GM concentrations, eGFR and side effects, were observed. Data are presented as mean ± standard deviation, except for months, which are described as median (interquartile range).

## Results

### Patient demographics

Five men and nine women, aged 69.5 ± 11.0 years (range 49–87 years old), developed PJIs after TKA during the study period and all were eligible for this study (Table 1). No patients were excluded or dropped out during follow-up. Among the 14 patients with PJIs, 5 were diagnosed with acute infection and 9 with chronic infection. The follow-up period was 18.4 (15.2–21.1) months.

**Table 1:** Demographic and surgical details of patients with periprosthetic joint infections (PJIs) following total knee arthroplasty (TKA) treated with a combination of continuous local antibiotic perfusion (CLAP).

Case	Age (years)	Sex	Affected side	Comorbidity	Body mass index (kg/m^2^)	Smoking status (Brinkman index)	Acute/chronic infection	Previous TKA surgery (primary/revision)	Time from TKA (days)	Culture	Time from recent infection diagnosis to surgery (days)	Surgery	CRP negative achievement (days from surgery)	Time to 2^nd^ surgery (days)	Follow-up period (months)	Success /failure
1	86	F	R	HT	29.4	Nonsmoker	Acute	Primary	29	Negative	0	DAIR, insert exchange	92		39.6	Success
2	77	M	L	–	24.1	Nonsmoker	Acute	Primary	204	MSSA	11	DAIR, insert exchange	64		17.5	Success
3	77	F	L	AP, HT, DLP, DM, asthma, GERD	19.7	Nonsmoker	Acute	Primary	62	MSSA, *Staphylococcus haemolyticus* (MRS)	1	DAIR, insert exchange	71		14.2	Success
4	87	M	R	RA, HT, ARF, prosthesis cancer	25.1	Nonsmoker	Acute	Primary	623	*Escherichia coli*	1	DAIR, insert exchange	126		1.49	Success
5	70	F	R	DM, HT, DLP	27.6	Nonsmoker	Acute	Revision	21	*Staphylococcus epidermidis*	3	DAIR, insert exchange	46		11.8	Success
6	49	F	L	RA	21.3	Nonsmoker	Chronic	Primary	4113	*Staphylococcus epidermidis*	416	Implant removal, cement spacers, debridement	14	180	21.2	Success
7	61	F	R	–	38.7	Nonsmoker	Chronic	Primary	1021	MSSA	17	Implant removal, cement spacers, debridement	32	168	16.5	Success
8	73	F	L	DM, HT, DLP	24.9	Ex-smoker (200)	Chronic	Primary	2616	MAC	27	Implant removal, cement spacers, debridement	27		20.3	Success
9	69	F	L	RA, hypothyroidism	17.7	Nonsmoker	Chronic	Primary	204	MSSA, Candida	91	Implant removal, cement spacers, debridement	159	112	40.5	Success
10	71	F	R	–	29.1	Nonsmoker	Chronic	Primary	482	MSSA	72	Implant removal, cement spacers, debridement	168	148	39.9	Success
11	74	M	L	HT, DLP	28.1	Ex-smoker (1,600)	Chronic	Revision	1647	*Staphylococcus lugdunensis*	63	Implant removal, cement spacers, debridement	29	133	20.7	Success
12	58	F	R	Sjogren disease, DM, RA, asthma, HT, DLP	25.2	Smoker (760)	Chronic	Revision	316	*Staphylococcus epidermidis*	193	Implant removal, cement spacers, debridement	102	105	14.2	Success
13	70	M	L	HT, lung cancer, lymphedema, pulmonary fibrosis	23.5	Smoker (760)	Chronic	Primary	1844	*Staphylococcus lugdunensis*	5	DAIR, insert exchange	91		16.2	Failure
	71	M	L				Chronic	Primary		*Staphylococcus lugdunensis*	13	Implant removal, cement spacers, debridement	62		20.7	Success
14	51	M	L	–	22.5	Smoker (1,120)	Chronic	Primary	247	*Staphylococcus hominis*	5	DAIR, insert exchange	71		19.4	Failure
	52	M	L				Chronic	Primary		*Bacillus* species	28	Implant removal, cement spacers, debridement	56		14.0	Success

HT, hypertension; AP, angina pectoris; DLP, dyslipidemia; DM, diabetes mellitus; GERD, gastroesophageal reflux disease; ARF, acute renal failure; RA, rheumatoid arthritis; MSSA, methicillin-sensitive *Staphylococcus aureus*; MRS, methicillin-resistant *Staphylococcus*; MAC, *Mycobacterium avium* complex; DAIR, debridement, antibiotics and implant retention; CRP, C-reactive protein.

### Treatment and related outcomes

CLAP therapy combined with DAIR was performed for all five patients with acute PJIs, among whom one underwent revision TKA and the others underwent primary TKA. The period from the final TKA was 2.1 (1.0–6.8) months, whereas the period from the diagnosis of PJIs to surgery was 1 (1–3) day. Four knees had positive cultures and one knee had a negative culture: MSSA with methicillin-resistant *Staphylococcus haemolyticus* (one knee), *Escherichia coli* (one knee), *Staphylococcus epidermidis* (one knee), and *Staphylococcus aureus* (one knee). The follow-up period was 14.9 (14.2–17.5) months. All patients with acute infection successfully preserved their implants.

Among the nine knees with chronic PJIs, seven underwent two-stage revision surgery, whereas the remaining two were treated with DAIR at the patients’ request. However, these two patients failed to achieve infection control and subsequently underwent additional two-stage revision surgery. Seven of the nine patients with PJIs who underwent two-stage revision surgery developed PJIs after primary TKA, whereas the remaining two knees, which underwent DAIR and subsequently failed, developed PJIs after revision TKA. The period from TKA was 34.0 (10.5–77.8) months, whereas the period from the first diagnosis of infection to surgery (performing CLAP) was 2.1 (0.6–3.0) months. All knees with chronic PJIs after TKA had positive cultures for the following pathogens: *Staphylococcus lugdunensis* (two knees), MSSA (two knees), MSSA with *Candida* (one knee), *Staphylococcus epidermidis* (two knees), *Mycobacterium avium* complex (one knee), and *Bacillus* species (one knee). The follow-up period was 20.3 months (range 16.5–21.2 months). At the final follow-up, all patients achieved infection control. No loosening of the implants was observed, and all patients were able to walk and perform their daily routines at home.

### CLAP outcomes and side effects

All 14 patients were able to continue CLAP for 2 weeks. No drain-related problems, such as obstruction, leakage, or drain site infection, were observed. Serum GM concentrations peaked within a week in almost all patients. Four patients had serum GM concentrations exceeding 2.0 μg/mL (Table 2). GM concentrations decreased in all patients after the dosage was reduced. The mean eGFR was 75.7 ± 31.2 mL/min/1.73 m^2^ preoperatively and 72.9 ± 24.3 mL/min/1.73 m^2^ at the final follow-up. No side effects of GM, such as renal function failure or ototoxicity, were noted throughout the treatment and follow-up period.

**Table 2:** Blood test results for renal function and gentamicin (GM) concentration.

Case	Preoperative eGFR (mL/min/1.73 m^2^)	Final eGFR (mL/min/1.73 m^2^)	Maximum serum GM concentration (μg/mL)	The day serum GM concentration reached maximum (day)	Maximum drain GM concentration (μg/mL)	Success/failure
1	72.4	57.0	0.5	7	590.0	Success
2	51.7	71.9	0.6	6	853.5	Success
3	92.0	96.4	1.2	3	840.0	Success
4	7.0	30.2	2.1	3	80.0	Success
5	118.0	99.0	1.0	7	730.0	Success
6	131.4	127.1	1.0	3	1180.0	Success
7	68.3	81.5	0.6	1	303.0	Success
8	87.4	70.6	0.3	7	390.0	Success
9	99.5	70.2	1.0	14	1090.0	Success
10	75.1	69.9	2.2	7	567.0	Success
11	56.4	51.1	2.8	1	730.0	Success
12	118.6	109.2	0.9	3	1120.0	Success
13	45.3	46.8	3.4	3	2050.0	Failure
	42.8	51.9	1.9	1	315.3	Success
14	79.0	71.8	1.0	1	390.0	Failure
	66.8	72.7	0.7	5	979.2	Success

eGFR, estimated glomerular filtration rate.

## Discussion

In this study, we reported the short-term outcomes of CLAP therapy for patients with PJIs after TKA. All patients with acute PJIs were able to retain their implants with CLAP therapy combined with DAIR. Among the nine patients with chronic PJIs, two who were initially treated with one-stage surgery and CLAP therapy failed to retain their implants, but all nine underwent revision surgery with CLAP therapy and achieved infection control. Although four patients had GM concentrations exceeding 2.0 μg/mL, no side effects of GM, such as renal function failure or ototoxicity, were observed throughout the follow-up periods.

Radical DAIR has been the standard therapy for acute PJIs [15, 16]. However, the success rate of DAIR depends on the toxicity of the bacteria. A previous review on DAIR for acute PJI after TKA reported success rates ranging from 16% to 60%, highlighting that acute PJI remains a challenging pathology [8, 17]. DAIR is often combined with continuous irrigation or local antibiotic treatment, resulting in success rates ranging from 73% to 88% [12, 18, 19]. In the present study, all patients with acute PJIs who underwent CLAP therapy combined with DAIR were able to retain their metal implants, indicating that CLAP therapy with DAIR may have superior effects on acute PJIs compared to conventional DAIR.

Revision surgery remains the standard strategy for chronic PJIs. Several reports on revision arthroplasties for chronic PJIs indicate success rates ranging from 72% to 93% [8, 20]. Among patients who developed chronic infections after TKA, all seven patients who underwent two-stage revision surgery with CLAP therapy successfully completed revision TKA without recurrence of infection or any complications. These excellent results suggest that CLAP therapy can be an effective treatment approach for chronic PJIs.

GM plays an important role in CLAP therapy, and the increased success rate of CLAP combined therapy for PJIs can be attributed to several reasons. First, CLAP therapy can provide sufficient local concentrations of GM while allowing for monitoring to control levels. Second, the effects of GM are concentration-dependent [21]. Third, high concentrations of GM are bactericidal for drug-resistant microbes, including methicillin-resistant *Staphylococcus aureus* (MRSA) [13]. Additionally, high concentrations of GM are more effective against biofilms than other antibiotics [12, 13]. High positivity rates for MRSA cultures (24%–82%) and biofilm formation are characteristic of PJIs [22, 23]. MRSA has been associated with a greater PJI treatment failure rate than other pathogens [24]. The minimum inhibitory concentration (MIC) of GM against MRSA has been reported to be 0.06–64 μg/mL, whereas the minimum biofilm eradication concentration (MBEC) has been estimated at 1–256 μg/mL [25]. Our study demonstrated a mean GM concentration in drainage fluid of 763.0 μg/mL, despite maintaining serum GM concentrations below 2.0 μg/mL. These results suggest that CLAP therapy can provide adequate GM concentrations for MRSA and its biofilms. The concentration required to eliminate biofilms (MBEC) is significantly higher than the MIC, making it infeasible to achieve MBEC through intravenous infusion due to the risk of systemic complications [13]. One study reported their experience with CLAP therapy in six patients with PJIs following TKA [26]. Their surgical approach was consistent with ours, and they documented successful outcomes in all patients without recurrent infection. Conversely, another study evaluated six patients who underwent DAIR combined with CLAP therapy for chronic PJIs: although the initial success rate of CLAP therapy was 67%, all patients ultimately retained their components [27]. Our own experience revealed two patients who failed to retain their implants with CLAP therapy combined with DAIR, both involving chronic PJIs. This underscores the limitations of CLAP therapy in conjunction with DAIR, and we should not overestimate its efficacy. Although CLAP therapy shows synergistic effects with DAIR for acute PJIs and with staged revision surgery for chronic PJIs, it does not always guarantee effectiveness for chronic PJIs. We emphasize that overconfidence in the efficacy of CLAP therapy may not benefit patients. Selecting an appropriate combined treatment strategy based on the infection status is crucial for achieving successful PJI outcomes, although further studies are needed to define these conditions. Our study included a larger number of patients than previous studies; however, additional cases are still necessary for future research. We also addressed more delayed infection PJIs, which may have contributed to these failures. Alongside therapeutic outcomes, we monitored GM concentrations to safely administer CLAP therapy, providing crucial information for this treatment approach.

Serum GM concentrations peaked within 7 days in almost all patients, consistent with findings reported previously [21]. To prevent side effects, GM concentrations were monitored every 2–3 days. Consequently, there were five patients (35.7%) who temporarily exhibited serum GM concentrations exceeding 2.0 μg/mL. All patients with serum GM concentrations over 2.0 μg/mL had an eGFR lower than 70 mL/min/1.73 m^2^ at the time of surgery (mean GFR was 50.1 ± 23.8 mL/min/1.73 m^2^). However, none suffered from ototoxicity or renal failure. Thus, frequent monitoring was crucial to prevent the side effects of CLAP therapy. Additionally, the amount of GM should be carefully controlled and may need to be reduced for patients with preoperative low eGFR.

This study has some limitations worth noting. First, as a retrospective single-arm study with a small sample size and a short follow-up period, it may contain hidden biases. The low incidence of PJIs at our institution due to various prophylactic treatments and that the recent introduction of CLAP therapy made it challenging to obtain samples over a longer follow-up period [28]. Therefore, we must consider this potential bias when concluding the true effectiveness of CLAP therapy, and future studies should ideally include a control group for comparison. Second, the background characteristics varied among patients, complicating comparisons of therapeutic effects. Nonetheless, this study is significant in reporting the outcomes and risks of CLAP therapy for both acute and chronic PJIs. The success rates and GM concentrations observed during CLAP therapy can provide valuable insights for future PJI treatments. Finally, this study did not assess the relationship between the overall success rate of treatment for PJIs, medical costs, and the risk of disability. Although we reported a 100% overall success rate, it is important to establish a therapy that is both less invasive and more cost-effective for clinical application while maintaining high success rates. In Japan, the healthcare system largely covers the medical costs associated with CLAP therapy. However, we believe that requiring patients to remain connected to syringe pumps with their legs immobilized for 2 weeks could be highly inconvenient and may not be practical within the healthcare systems of certain countries. Therefore, we recommend considering medical costs when applying CLAP therapy, in accordance with each country’s healthcare system and adjusting postoperative care accordingly.

In conclusion, this study elucidated the short-term outcomes of CLAP therapy for acute and chronic PJIs after TKA. Despite the need to monitor for ototoxicity and renal failure, CLAP therapy was safe and may be effective when combined with conventional treatments for acute and most chronic PJIs.

## Data Availability

The datasets used and analyzed during the current study are available from the corresponding author on reasonable request.
